# Metabolomics in Infectious Diseases and Vaccine Response: Insights into Neglected Tropical and Non-Neglected Pathogens

**DOI:** 10.3390/idr18010010

**Published:** 2026-01-12

**Authors:** Mahbuba Rahman, Hasbun Nahar Hera, Urbana Islam Barsha

**Affiliations:** Department of Biochemistry and Microbiology, North South University, Dhaka 1229, Bangladesh; hasbun.hera@northsouth.edu (H.N.H.); islam.barsha@northsouth.edu (U.I.B.)

**Keywords:** vaccine, neglected tropical disease, non-neglected tropical disease, metabolomics, infectious diseases

## Abstract

Background/objectives: Metabolomics has emerged as a powerful systems-biology tool for deciphering dynamic metabolic alterations occurring during infectious diseases and following vaccination. While genomics and proteomics provide extensive molecular and regulatory information, metabolomics uniquely reflects the biochemical phenotype associated with infection, immune activation, and immunometabolic reprogramming. The objective of this review is to provide an integrated analysis of metabolomics applications across both neglected tropical diseases (NTDs) and non-NTD pathogens, highlighting its dual role in biomarker discovery and vaccine response evaluation. Methods: A comprehensive literature-based synthesis was conducted to examine metabolomic studies in infectious diseases and vaccinology. Metabolic perturbations associated with specific pathogens, as well as vaccine-induced metabolic changes and correlates of immune responses, were systematically analyzed and compared across NTD and non-NTD contexts. Results: Distinct pathogen- and vaccine-associated metabolic signatures were identified, reflecting alterations in glycolysis, amino acid metabolism, lipid remodeling, and immunoregulatory pathways. Comparative analysis revealed both shared and disease-specific metabolic biomarkers across NTDs and non-NTD infections. Importantly, vaccine-related metabolic correlates were shown to mirror immune activation states and, in some cases, predict immunogenicity and response durability. Conclusions: This review bridges metabolomics research in infectious disease pathogenesis and vaccine immunology across the NTD and non-NTD spectrum. By integrating these domains, it introduces the concept of “metabolic immuno-signatures” as predictive and translational tools for evaluating vaccine efficacy and immune response outcomes.

## 1. Introduction

Infectious diseases continue to impose a substantial global health burden, with both non-neglected (e.g., tuberculosis, influenza, HIV, COVID-19) and neglected tropical diseases (e.g., malaria, leishmaniasis, schistosomiasis, Chagas disease) responsible for millions of deaths annually [1]. The World Health Organization classifies more than 20 conditions as NTDs, collectively affecting over one billion people in tropical and subtropical regions. At the same time, highly transmissible non-NTD pathogens continue to drive recurrent epidemics and pandemics, placing significant pressure on healthcare systems and posing ongoing challenges for vaccine development and deployment [2]. Vaccinology—the science of vaccine development and implementation—has been a cornerstone of public health, contributing to the eradication or effective control of numerous infectious diseases [3]. Conventional vaccines are designed to elicit protective immune responses against bacterial, viral, or parasitic pathogens, thereby preventing diseases such as smallpox, polio, and measles [4]. This is typically achieved using live attenuated or inactivated organisms, or purified pathogen components such as proteins or polysaccharides that stimulate immune recognition without causing disease [3].

Despite these successes, significant limitations remain. Vaccine-induced protection can vary widely across populations due to host-related factors including genetic background, age, comorbidities, nutritional status, and environmental exposures. The traditional “one-size-fits-all” paradigm in vaccinology fails to adequately account for this interindividual variability, often resulting in reduced efficacy among vulnerable groups such as older adults, immunocompromised individuals, and populations with distinct genetic or metabolic profiles [3,4]. Additional challenges arise from rapidly evolving pathogens such as influenza viruses and HIV, as well as the emergence of novel infectious agents exemplified by SARS-CoV-2.

Improving existing vaccines is therefore critical for enhancing disease control and preventing outbreaks of re-emerging infectious diseases [5,6]. The rational design and optimization of vaccines require robust methods for monitoring vaccine efficacy, which depend on the identification and validation of reliable biomarkers. In the context of infectious diseases, biomarkers are defined as objectively measurable characteristics that indicate pathogenic processes, normal biological states, or biological responses to therapeutic interventions. Within vaccinology, such biomarkers serve as vaccine-specific signatures and must be statistically distinguishable between vaccinated and unvaccinated individuals while reflecting immunization-induced biological responses [5,6].

The host response to vaccination can be comprehensively characterized by systems vaccinology, which integrates multi-omics technologies, including genomics, transcriptomics, proteomics, and metabolomics. These high-throughput approaches enable the assessment of differential gene expression in peripheral blood mononuclear cells (PBMCs), whole blood, and specific immune cell subsets through transcriptomic analyses, thereby providing an integrated view of the cellular and physiological responses elicited following vaccine administration [7]. However, gene expression–based analyses primarily capture relatively static or upstream regulatory responses and do not fully capture the dynamic biochemical activities that underpin immune effector function, [3].

In contrast, metabolomics directly interrogates downstream biochemical processes by quantitatively profiling low-molecular-weight metabolites (<1500 Da), offering a dynamic and integrative readout of immune cell activation, immunometabolic reprogramming, and host–pathogen interactions in response to vaccination or infection [8,9]. Advances in analytical platforms such as nuclear magnetic resonance (NMR) spectroscopy, liquid chromatography–mass spectrometry (LC–MS), and gas chromatography–mass spectrometry (GC–MS) have enabled the systematic identification of vaccine- and disease-associated metabolic signatures [10]. These approaches have yielded metabolomic biomarkers capable of distinguishing infected from healthy individuals, stratifying disease stages, and predicting vaccine responsiveness. Importantly, such biomarkers have been reported across both neglected tropical diseases (NTDs) and non-NTD infectious diseases, providing novel mechanistic insights into pathogenesis, immune regulation, and vaccine-induced protection [11].

This review advances the field by presenting the first comparative synthesis of metabolomics data encompassing both neglected and non-neglected infectious pathogens, with a specific focus on linking metabolic biomarkers to infection and vaccine response outcomes. The novelty of this work lies in its integration of pathogen-specific metabolic alterations with shared immunometabolic pathways and its proposal of a translational framework for precision vaccinology informed by metabolomic signatures.

## 2. Metabolomics as Disease Biomarker in Infectious Diseases

### 2.1. Non-Neglected Infectious Diseases (NTDs)

Metabolomics has been widely applied to non-NTD infections such as tuberculosis (TB), malaria, COVID-19, and influenza. These diseases account for a large portion of global morbidity and mortality and exhibit distinct metabolic signatures associated with pathogen virulence and immune evasion (Table 1).

#### 2.1.1. Tuberculosis

*Mycobacterium tuberculosis* (family *Mycobacteriaceae*) remains one of the world’s top infectious killers, causing nearly 1.3 million deaths annually. Pathogenesis involves intracellular survival within macrophages and metabolic adaptation under hypoxia. Metabolomic studies reveal elevated lactate, decreased arginine, and enhanced triacylglycerols reflecting host lipid metabolism reprogramming [12]. Plasma metabolite panels distinguishing active from latent TB include decreased tryptophan and increased kynurenine, signaling immune activation [15].

#### 2.1.2. COVID-19

SARS-CoV-2 (family *Coronaviridae*) causes severe respiratory and systemic illness. Global morbidity exceeded 700 million infections by 2024. LC–MS-based analyses show perturbations in amino acid, bile acid, and lipid metabolism. Reduced sphingolipids and elevated kynurenine/tryptophan ratios correlate with severe disease and cytokine storm [12,16]. In addition, lactate levels were high in severe patients [17].

#### 2.1.3. HIV

HIV-1 (family *Retroviridae*) induces chronic immune activation. Metabolomics reveals increased glutaminolysis and T-cell exhaustion metabolites [5,12].

#### 2.1.4. Influenza

In Influenza A infection (*Orthomyxoviridae*), decreased level of carnitine was associated with poor prognosis of the infection [18].

Both HIV and influenza infections display metabolic convergence in energy and redox pathways [19].

### 2.2. Neglected Tropical Diseases (NTDs)

NTDs remain a major cause of morbidity and mortality in tropical and subtropical regions [20]. These infections are associated with chronic immune activation, oxidative stress, and metabolic dysregulation, all of which are detectable through metabolic signatures (Table 1).

#### 2.2.1. Malaria

*Plasmodium falciparum* (family *Plasmodiidae*) causes ~240 million cases annually. Pathogenesis results from parasite invasion of red blood cells, leading to hemolysis and severe anemia. LC–MS analyses of plasma show increased lysophosphatidylcholines, lactate, and hypoxanthine, indicating tissue hypoxia and purine salvage. Urinary metabolites such as pipecolate and phenylacetylglutamine serve as diagnostic biomarkers [10].

#### 2.2.2. Leishmaniasis

*Leishmania donovani* (family *Trypanosomatidae*) infects macrophages, causing visceral leishmaniasis (VL) with high mortality if untreated. Metabolomics reveals suppressed tricarboxylic-acid (TCA) intermediates and enhanced polyamine metabolism, supporting parasite replication [14]. Distinct serum markers (ornithine, putrescine) differentiate active VL from post-treatment states.

#### 2.2.3. Schistosomiasis

*Schistosoma mansoni* (family *Schistosomatidae*) affects over 230 million individuals worldwide. Pathogenesis involves granulomatous inflammation in the liver and intestines. Metabolomics shows increased bile acids, taurine, and oxidative-stress products [11]. These signatures serve as indicators of fibrosis progression and therapeutic response.

#### 2.2.4. Chagas Disease

*Trypanosoma cruzi* (family *Trypanosomatidae*) causes chronic cardiomyopathy in Latin America. Altered plasma lipids and reduced sphingomyelins reflect myocardial energy depletion. Early detection via LC–MS biomarkers (acylcarnitines, lysophospholipids) could improve disease monitoring [21].

#### 2.2.5. Dengue Fever

Although dengue is sometimes categorized as an NTD, it also bridges non-NTD research due to its global burden. The disease, caused by *Dengue virus* (*Flaviviridae*), leads to high fever and in severe cases, hemorrhagic manifestations. Metabolomic studies using serum and plasma have identified biomarkers including elevated lactate, pyruvate, and sphingolipids, which reflect endothelial dysfunction and cytokine storm. Notably, increased levels of lysophospholipids and reduced tryptophan indicate immune–metabolic reprogramming associated with severe dengue [5,12].

## 3. Metabolomics in Vaccine Response

Vaccination triggers complex immunometabolic changes that determine both the magnitude and durability of immune protection. Metabolomics, the large-scale analysis of small molecules within cells, tissues, or biofluids has emerged as a powerful systems-level approach for decoding these molecular signatures of immunity. By profiling metabolites before and after vaccination, researchers can identify metabolic pathways that correlate with antigen processing, antibody production, and cellular immune responses (Table 2) [22].

### 3.1. Immunometabolic Remodeling Following Vaccination

Upon immunization, host cells, particularly dendritic cells, macrophages, and lymphocytes undergo rapid metabolic reprogramming to meet energetic and biosynthetic demands. Early activation of glycolysis and the tricarboxylic acid (TCA) cycle provides ATP and intermediates for biosynthetic pathways [27]. Metabolomic analyses have shown that effective vaccine responders exhibit higher glycolytic flux and enhanced amino acid metabolism, reflecting robust immune cell activation [28].

For instance, in influenza vaccination, plasma metabolomics revealed increased levels of pyruvate, lactate, and TCA intermediates in high responders compared to non-responders, suggesting a link between metabolic activity and antibody titers [24]. Similarly, lipidomic shifts involving phosphatidylcholines and sphingolipids have been observed in responders to the hepatitis B vaccine, indicating membrane remodeling necessary for lymphocyte activation [28].

### 3.2. Metabolomics as a Predictive Tool for Vaccine Efficacy

Pre-vaccination metabolic signatures can serve as predictors of vaccine responsiveness. For example, tryptophan and its downstream metabolite kynurenine regulated by the enzyme indoleamine 2,3-dioxygenase (IDO) have emerged as important immunomodulators. Elevated kynurenine to tryptophan ratios before vaccination correlate with suppressed immune activation, suggesting that individuals with high basal IDO activity may exhibit weaker vaccine-induced responses [9].

In yellow fever vaccination, one of the best-studied live attenuated vaccines, metabolomics revealed strong correlations between early lipid metabolism and long-term CD8^+^ T-cell responses. The upregulation of lysophosphatidylcholine (LPC) species was linked with innate immune activation and antigen presentation [29]. These findings demonstrate that lipid metabolites not only reflect immune status but may actively modulate it through signaling pathways involving toll-like receptors and inflammasomes.

### 3.3. Metabolomics in Vaccine Response to Non-NTD Pathogens

#### 3.3.1. Influenza and COVID-19 Vaccines

Metabolomic profiling after influenza vaccination has revealed a consistent activation of glycolysis and the pentose phosphate pathway, essential for nucleotide synthesis and antibody gene transcription [24]. In mRNA-based COVID-19 vaccines, studies have shown changes in arginine and glutamine metabolism linked to immune cell proliferation and interferon signaling [23]. Elevated levels of carnitine and fatty acid oxidation intermediates were found in high responders, indicating enhanced mitochondrial metabolism and oxidative phosphorylation that supports long-lived plasma cell development [24].

#### 3.3.2. Tuberculosis (BCG) Vaccine

In *Mycobacterium bovis* BCG vaccination, metabolomics studies have revealed alterations in tryptophan-kynurenine metabolism and glutathione redox balance [15]. These metabolites serve as early correlates of vaccine-induced trained immunity where innate immune cells develop a heightened response to secondary infections through epigenetic and metabolic reprogramming [5].

#### 3.3.3. Hepatitis B Vaccine

In hepatitis B vaccine responders, specific phospholipid and sphingolipid metabolites were found to correlate with high antibody titers and memory B cell formation [30]. In contrast, non-responders often display impaired one-carbon metabolism, affecting methylation reactions and epigenetic regulation of immune genes [5].

### 3.4. Metabolomics in Vaccine Response to Neglected Tropical Diseases (NTDs)

#### 3.4.1. Dengue Virus

Dengue infection induces profound metabolic reprogramming involving tryptophan metabolism, lipid biosynthesis, and energy production [31]. Vaccine-induced immunity (e.g., Dengvaxia and TAK-003) also triggers metabolic pathways similar to natural infection but with distinct regulatory patterns. Metabolomic profiling post-vaccination has identified elevated kynurenine and glycerophospholipids, associated with interferon-gamma production and balanced Th1/Th2 responses [32].

#### 3.4.2. Leishmaniasis

Experimental vaccines against *Leishmania donovani* have been associated with metabolic reprogramming in macrophages and T cells, with increased flux through glycolysis and arginine metabolism [26]. Arginine availability is crucial for nitric oxide synthesis, a key effector molecule in parasite clearance. Metabolomics-based biomarker studies have shown that successful vaccination correlates with elevated polyamine metabolism and lipid remodeling, reflecting macrophage activation states [25].

#### 3.4.3. Schistosomiasis

In *Schistosoma mansoni* vaccine trials, serum metabolomics revealed changes in acylcarnitines and amino acid catabolites post-immunization, which correlated with protective antibody titers [11]. Perturbations in the TCA cycle intermediates (succinate, fumarate) indicate metabolic rewiring toward proinflammatory macrophage polarization—an essential component of effective immunity against schistosome larvae [11].

#### 3.4.4. Trypanosomiasis and Chagas Disease

Metabolomic studies in *Trypanosoma cruzi* and *T. brucei* infections have identified changes in host lipid and nucleotide metabolism, reflecting immune evasion strategies [33]. Vaccine development efforts have used these insights to design immunogens that mimic metabolic environments promoting Th1 responses. Elevated succinate and fumarate were observed as biomarkers of mitochondrial activation in successful immunizations [34].

## 4. Comparative Metabolic Themes Across Pathogens

Comparative metabolomic analyses across neglected tropical diseases (NTDs) and non-NTD infectious diseases reveal a complex interplay between conserved host immune responses and pathogen-specific metabolic adaptations. Despite major differences in pathogen biology—ranging from intracellular bacteria and viruses to extracellular parasites—several metabolic pathways consistently emerge as central regulators of disease progression and vaccine-induced immunity. These shared pathways form a core immunometabolic framework, while disease-specific metabolites reflect unique pathogenic strategies, tissue tropism, and host–pathogen interactions.

### 4.1. Conserved Metabolic Biomarkers Across NTD and Non-NTDs

One of the most consistently observed metabolites across both NTD and non-NTD infections is lactate, a hallmark of enhanced glycolytic flux. Elevated lactate levels have been reported in tuberculosis, malaria, and COVID-19, reflecting hypoxia, mitochondrial dysfunction, and immune cell activation during infection [12,17]. Beyond serving as a passive by-product of anaerobic metabolism, lactate actively modulates immune responses by shaping macrophage polarization, suppressing cytotoxic T-cell function, and influencing cytokine secretion. Its consistent association with disease severity across diverse pathogens underscores lactate as a robust, cross-cutting biomarker of inflammatory burden and immune dysregulation [35].

Another conserved metabolic axis is the tryptophan–kynurenine pathway, which plays a critical role in immune tolerance and regulation. Increased kynurenine levels have been documented in both NTDs such as malaria and non-NTD infections including tuberculosis and HIV [12]. Indoleamine-2,3-dioxygenase (IDO)-mediated tryptophan catabolism limits T-cell proliferation while promoting regulatory immune phenotypes, representing a common immune evasion strategy employed by diverse pathogens. Importantly, modulation of this pathway has also been observed following vaccination, suggesting that kynurenine-related metabolites may serve as predictive biomarkers of vaccine responsiveness and immune durability [36].

Lipid metabolism constitutes another shared metabolic hallmark, especially in vaccine studies for virus and parasites. Lipid metabolic reprogramming has been linked to antigen presentation efficiency and antibody production, further highlighting its translational relevance [30,31,34].

Together, these conserved metabolic features reveal a unifying immunometabolic signature underlying host responses to infection and immunization.

### 4.2. NTD-Specific Metabolic Signatures Reflecting Parasitic Adaptation

In contrast to these shared biomarkers, several metabolites are predominantly associated with NTDs, reflecting the unique biology of parasitic pathogens. Hypoxanthine, for example, is a key biomarker in malaria and arises from the parasite’s reliance on host purine salvage pathways due to its inability to synthesize purines de novo. Elevated hypoxanthine levels not only indicate parasite burden but also contribute to oxidative stress and endothelial dysfunction, linking metabolism directly to malaria pathogenesis [13,37].

Similarly, polyamine metabolites such as ornithine and putrescine are strongly associated with leishmaniasis. These metabolites reflect parasite-driven manipulation of host arginine metabolism, diverting substrates away from nitric oxide production and thereby impairing macrophage-mediated parasite killing [14]. Such metabolic rerouting exemplifies how parasitic pathogens exploit host metabolic pathways to establish chronic infection.

Helminth infections, including schistosomiasis, exhibit distinct alterations in bile acids and taurine metabolism, linked to chronic inflammation, liver pathology, and modulation of the gut–liver–immune axis [11]. These NTD-specific signatures highlight metabolic pathways that are rarely dominant in viral or bacterial infections, emphasizing the importance of pathogen-contextualized metabolomic interpretation.

### 4.3. Non-NTD-Specific Metabolic Signatures in Viral and Bacterial Diseases

Non-NTD infectious diseases, particularly viral infections such as COVID-19, display distinct metabolic perturbations that are less prominent in NTDs. Bile acids, lysophospholipids, and sphingolipids have emerged as key discriminators of disease severity in COVID-19, reflecting systemic inflammation, immune cell trafficking, and disruption of host lipid signaling pathways [12]. These metabolites are closely linked to the gut–lung axis and have been proposed as biomarkers for stratifying patients based on clinical outcomes.

Vaccine studies targeting non-NTD pathogens further reveal metabolic pathways associated with rapid immune activation. For instance, serine biosynthesis and glycolytic intermediates are enriched following mRNA COVID-19 vaccination, supporting the high biosynthetic demands of proliferating T and B cells [23]. Such signatures are less frequently observed in NTD vaccine studies, reflecting differences in vaccine platforms, immune kinetics, and pathogen biology.

### 4.4. Vaccine-Associated Metabolic Signatures Across Disease Categories

Despite pathogen-specific differences, vaccine-induced metabolic reprogramming shows notable convergence across NTD and non-NTD contexts. Enhanced glycolysis and amino acid metabolism consistently correlate with T-cell activation, interferon-γ production, and antibody titers following vaccination against tuberculosis, malaria, influenza, and SARS-CoV-2 [15,23,24]. In NTD vaccines, additional emphasis is placed on arginine–nitric oxide metabolism, particularly in experimental leishmaniasis vaccines, where macrophage activation and parasite clearance depend on metabolic support for effector functions [25,26].

Table 3 shows comparison of both conserved and pathogen-specific immunometabolic biomarkers across NTD and non-NTD infections and vaccine responses.

## 5. Integrative Model: Metabolic Immuno-Signature

Metabolite-mediated control of immune cell subsets is now a defining feature of infection and vaccine responses:

- Effector T cells depend on glycolysis and glutaminolysis for rapid proliferation.
- Memory T cells utilize OXPHOS and FAO for long-term survival.
- Macrophages exhibit metabolic plasticity between glycolytic (M1) and oxidative (M2) states, influencing pathogen clearance versus tissue repair.
- B cells use lipid metabolism for antibody secretion and plasma cell differentiation [38].

Metabolomics allows mapping of these signatures, distinguishing protective immunity from immunopathology. For instance, dengue vaccine responders display balanced glycolytic and lipid metabolic pathways, whereas non-responders show excessive glycolysis leading to exhaustion [31].

**Figure 1:**
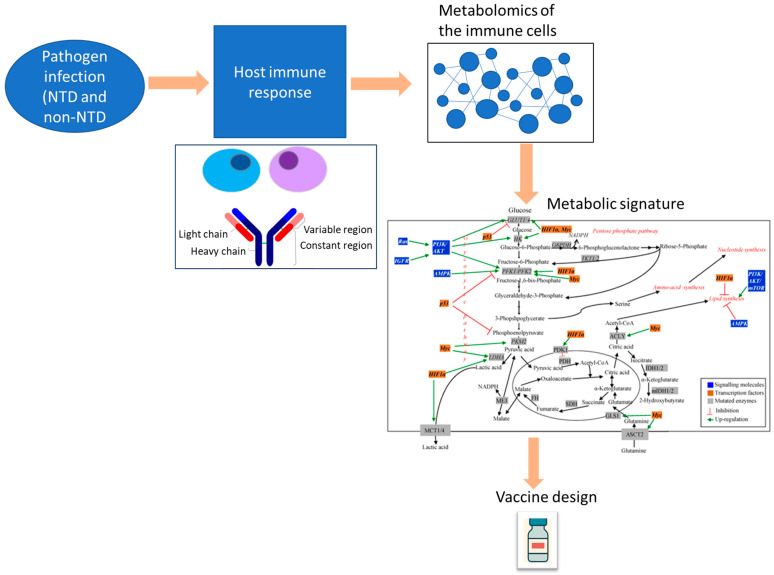
Figure showing application of metabolic signature for vaccine design. (This figure is modified from https://www.mdpi.com/2218-1989/5/4/571 (accessed on 24 November 2025), under Creative Common CC BY license).

Collectively, these findings form the conceptual basis of the “Metabolic Immuno-Signature” model (Figure 1). This framework proposes that immune protection depends on coordinated regulation of glycolysis, TCA intermediates, and lipid remodeling—fine-tuned by amino acid metabolism and epigenetic modulation. Pathogen-specific differences (e.g., *Leishmania* inducing arginine flux, SARS-CoV-2 activating FAO) generate distinct metabolic landscapes that influence vaccine outcomes.

Metabolomics thus provides a systems-level lens through which immune responses can be quantitatively predicted, enabling rational design of next-generation vaccines targeting specific metabolic nodes.

## 6. Challenges in Metabolomics for Infectious Diseases and Vaccine Response

While metabolomics has revolutionized the understanding of infectious diseases and vaccine immunology, its full translational potential is constrained by a series of technical, analytical, and biological challenges, which are particularly pronounced when comparing NTDs with globally prioritized infections like tuberculosis and COVID-19.

### 6.1. Technical and Analytical Variability

Methodological heterogeneity remains a major limitation in metabolomics studies. Variations in sample type (plasma, serum, urine, or tissue), collection timing, fasting status, storage conditions, and processing protocols can markedly influence metabolite profiles, thereby hindering reproducibility and cross-study comparison, especially in resource-limited NTD-endemic settings [39,40]. In addition, analytical platforms—including NMR, LC–MS, and GC–MS—differ in sensitivity, metabolite coverage, and reproducibility. While LC–MS offers broad coverage and high sensitivity, NMR provides superior reproducibility but lower sensitivity. These differences underscore the need for standardized pipelines for metabolite quantification, normalization, and cross-platform harmonization [10,41].

### 6.2. Data Complexity and Interpretation

Metabolomic datasets are inherently complex, characterized by high dimensionality and biological variability. A substantial proportion of detected features remain unidentified or ambiguously annotated, limiting biological interpretation [39,40]. Furthermore, integration of metabolomics with other omics layers remains underdeveloped in infectious disease research, particularly for NTDs where reference genomes and curated metabolomic databases are incomplete. Establishing causal relationships between metabolites and immune mechanisms requires complementary functional validation, isotopic tracing, or targeted metabolomics approaches [42].

### 6.3. Biological Variability and Host Factors

Host-related factors such as age, sex, diet, gut microbiota composition, and genetic background contribute significantly to baseline metabolic variability, complicating the identification of infection-specific signatures. In NTD-endemic regions, co-infections, malnutrition, and environmental exposures further confound metabolic profiles [43,44]. Additionally, strain-level pathogen heterogeneity influences metabolic outputs, highlighting the importance of integrating pathogen genotyping with metabolomic analyses [45].

### 6.4. Temporal and Spatial Dynamics

Metabolic responses to infection and vaccination are highly dynamic and context dependent. Acute immune activation, adaptive immune development, and localized tissue pathology generate temporal and spatial heterogeneity that may not be captured by cross-sectional sampling. Longitudinal and spatially resolved approaches are therefore essential but remain logistically challenging, particularly in low-resource settings [38,46].

### 6.5. Translational and Clinical Implementation Barriers

Despite the identification of numerous candidate metabolic biomarkers, few have progressed to clinical validation [47]. Key barriers include the lack of large multicenter studies, limited access to high-end analytical infrastructure in NTD-endemic regions, and regulatory and cost constraints associated with assay development. Diseases with limited commercial markets, such as leishmaniasis and schistosomiasis, are particularly affected. Addressing these challenges will require international consortia, technology transfer initiatives, and coordinated capacity-building programs [48].

### 6.6. Integration with Vaccine Research and Systems Immunology

In vaccine studies, metabolomics faces additional challenges due to the subtle and transient nature of post-vaccination metabolic changes, which require highly sensitive detection and precisely timed sampling [8]. Vaccine-induced metabolic responses often overlap with background immune activation from prior infections or environmental exposure, complicating the identification of vaccine-specific signatures. Although integration with systems vaccinology—encompassing genomics, transcriptomics, proteomics, and immune phenotyping—offers a powerful framework, it demands advanced computational tools, standardized metadata, and ethically complex longitudinal sampling strategies [49].

### 6.7. Gaps in Neglected Tropical Disease Research

Compared with non-NTD pathogens, NTDs remain underrepresented in metabolomics research due to limited funding, infrastructure, trained personnel, and biosafety constraints [50]. Most studies rely on small cohorts or animal models, limiting generalizability to human disease. Expanding metabolomics research in NTDs will require targeted funding mechanisms, regional research hubs, and sustained capacity-building efforts [42].

### 6.8. Data Sharing and Standardization Needs

The fragmentation of metabolomics data across platforms and institutions limits reproducibility and comparative analyses. Although repositories such as MetaboLights, GNPS, and Metabolomics Workbench exist, their adoption in infectious disease research remains inconsistent [51]. The development of pathogen-specific metabolomic databases and harmonized metadata standards—including infection stage, sample origin, and demographic variables—would substantially enhance data integration. Emerging artificial intelligence and machine learning approaches offer promise for extracting predictive signatures from complex datasets [52].

### 6.9. Limitations of Metabolomics for Determining Vaccine Efficacy

Despite its value in systems vaccinology, metabolomics alone is insufficient to robustly determine vaccine efficacy. Vaccine-induced metabolic perturbations are often transient and highly time dependent, frequently resolving before durable adaptive immune responses are established [8,9]. Moreover, metabolic changes often reflect generalized immune activation rather than antigen-specific protective immunity, limiting their utility as direct correlates of protection [3]. Interindividual variability driven by host factors and endemic immune activation further complicates biomarker discovery, particularly for NTD vaccines [4,24].

## 7. Conclusions

Metabolomics has emerged as a powerful tool in infectious disease research by providing direct insight into the biochemical processes that govern host–pathogen interactions and vaccine-induced immunity. However, to fully capture the complexity of immune responses and metabolic remodeling during infection and vaccination, metabolomics must be integrated with complementary omics technologies. By reflecting the downstream metabolic consequences of genomic and immunological regulation, and particularly when combined with transcriptomic and proteomic data, metabolomics enables a systems-level understanding that links immune function with dynamic metabolic reprogramming.

This review highlights an integrative comparison of neglected tropical diseases (NTDs) and non-NTD infections, revealing both shared and disease-specific metabolic signatures across parasites, helminths, viruses, and bacteria. Conserved perturbations in tryptophan–kynurenine, arginine–nitric oxide, and lipid pathways underscore common mechanisms of immune modulation, while pathogen-specific metabolites reflect distinct strategies of metabolic exploitation and persistence.

From a translational perspective, metabolomics-based biomarkers show promise for disease stratification, prognosis, and vaccine monitoring Table 4. This is particularly relevant for NTDs, where accessible metabolic biomarkers could address diagnostic gaps, while in non-NTD infections such as tuberculosis and COVID-19, metabolic signatures increasingly inform disease severity and treatment response. Vaccine metabolomics further demonstrates that vaccination-induced metabolic rewiring correlates with immune outcomes, supporting the use of metabolic signatures as early predictors of vaccine responsiveness.

Despite its promise, clinical translation is limited by challenges in standardization, inter-individual variability, and causal interpretation. Integration with multi-omics data, machine learning, and longitudinal study designs will be essential to advance metabolomics toward precision diagnostics and personalized vaccine strategies across both neglected and non-neglected infectious diseases.

## Figures and Tables

**Table 1:** Representative metabolomic platforms, sample types, and metabolites detected in NTD and non-NTD infections.

Disease	Pathogen (Family)	Sample Type	Platform	Key Metabolites	Brief Summary of Metabolomic Findings	Reference
Tuberculosis	*Mycobacterium tuberculosis* (*Mycobacteriaceae*)	Plasma, sputum	LC–MS	Kynurenine, lactate, TAGs	Reflects chronic immune activation, enhanced glycolysis, and lipid remodeling associated with macrophage reprogramming and mycobacterial persistence; useful for distinguishing active disease and monitoring treatment response.	[12]
COVID-19	SARS-CoV-2 (*Coronaviridae*)	Serum	LC–MS, NMR	Sphingolipids, bile acids	Indicates systemic immune dysregulation, endothelial dysfunction, and hepatic involvement; metabolic signatures correlate with disease severity and inflammatory burden.	[12]
HIV-1	*Retroviridae*	Plasma	LC–MS	Glutamine, kynurenine	Highlights sustained immune activation and T-cell dysfunction driven by chronic inflammation and altered tryptophan metabolism; relevant for disease progression and therapeutic monitoring.	[12]
Malaria	*Plasmodium falciparum* (*Plasmodiidae*)	Plasma, urine	LC–MS	3-hydroxybutyric acid, valine, hypoxanthine, lactate	Reflects increased nucleotide turnover and anaerobic glycolysis resulting from parasite replication and host hypoxia; correlates with parasite burden and disease severity.	[12,13]
Leishmaniasis	*Leishmania donovani* (*Trypanosomatidae*)	Serum	GC–MS	Ornithine, putrescine	Demonstrates dysregulated polyamine metabolism critical for parasite survival and macrophage function; provides insight into host–parasite metabolic crosstalk and disease progression.	[14]
Schistosomiasis	*Schistosoma mansoni* (*Schistosomatidae*)	Plasma	LC–MS	Bile acids, taurine	Indicates hepatobiliary dysfunction, oxidative stress, and chronic inflammation driven by egg-induced tissue pathology; useful as non-invasive biomarkers of liver involvement.	[11]

**Table 2:** Metabolomic correlates of vaccine response in NTD and non-NTD pathogens.

Vaccine	Pathogen	Platform	Key Metabolic Pathways	Associated Immune Outcome	Brief Summary of Metabolomic Insights	Reference
mRNA (COVID-19)	SARS-CoV-2	LC–MS	Glycolysis, amino acid metabolism	T-cell activation	Demonstrates rapid metabolic reprogramming toward aerobic glycolysis and one-carbon metabolism, supporting clonal expansion and effector differentiation of vaccine-induced T cells.	[23]
Influenza (TIV)	Influenza A	NMR	Phospholipid metabolism	Antibody titers	Highlights lipid remodeling and membrane biosynthesis required for B-cell activation and plasmablast differentiation, with phospholipid profiles correlating with humoral vaccine efficacy.	[24]
TB (M72/AS01E)	*Mycobacterium tuberculosis*	LC–MS	Tryptophan, kynurenine	IFN-γ production	Reveals enhanced amino acid utilization and lipid oxidation that support Th1 polarization and sustained IFN-γ secretion, key correlates of protective anti-tuberculosis immunity.	[15]
Leishmania vaccine (experimental)	*Leishmania donovani*	LC–MS	Glycolysis, arginine and nitric oxide metabolism	Macrophage activation	Reflects metabolic rewiring of macrophages toward nitric oxide production, enhancing parasite killing and supporting vaccine-induced cell-mediated immunity.	[25,26]

**Table 3:** Shared and Distinct Metabolic Biomarkers in NTD and Non-NTD Infectious Diseases and Vaccine Responses.

Category	Metabolic Biomarkers	Presence in NTD	Presence in Non-NTD	Biological/Immunological Significance	Representative Diseases/Vaccines (References)
Common metabolic biomarkers	Lactate	√	√	Indicator of hypoxia, enhanced glycolysis, and inflammatory burden; correlates with disease severity and immune activation	Tuberculosis, Malaria, COVID-19 [12]
	Kynurenine/tryptophan pathway metabolites	√	√	Reflects immune regulation via IDO activity, T-cell suppression, and modulation of vaccine-induced immunity	TB, Malaria, and HIV [12], Influenza vaccine [24]
	Lipid metabolism (TAGs, phospholipids)	√	√	Linked to membrane remodeling, antigen presentation, immune cell activation, and pathogen survival strategies	TB and COVID-19 [12], Influenza vaccine [24]
	Amino acid metabolism (glutamine, arginine)	√	√	Supports immune cell proliferation, macrophage activation, nitric oxide production, and cytokine synthesis	TB and COVID-19 [12], Leishmaniasis [14]
Predominantly NTD-associated biomarkers	Hypoxanthine	√	×	Marker of parasite-driven purine salvage, host energy stress, and oxidative damage	Malaria [13]
	Ornithine, putrescine (polyamines)	√	×	Reflects parasite-mediated diversion of arginine metabolism and impaired macrophage-mediated killing	Leishmaniasis [14]
	Taurine	√	×	Associated with helminth-induced bile acid metabolism, osmotic regulation, and chronic inflammation	Schistosomiasis [11]
Predominantly non-NTD-associated biomarkers	Bile acids	×	√	Reflect systemic inflammation and dysregulation of the gut–liver–immune axis	COVID-19 [12]
	Lysophospholipids, sphingolipids	×	√	Involved in viral entry, immune signaling, endothelial dysfunction, and severity stratification	COVID-19 [12]
	Amino acid biosynthesis intermediates	×	√	Supports rapid lymphocyte proliferation and effector differentiation following vaccination	mRNA COVID-19 vaccine [23]
Vaccine-specific metabolic signatures	Glycolysis intermediates	√	√	Predicts T-cell activation, IFN-γ production, and effector differentiation post-vaccination	TB (M72/AS01E) [15], COVID-19 mRNA vaccine [23]
	Nitric oxide-related metabolites	√	×	Associated with macrophage activation and intracellular parasite killing	Experimental Leishmania vaccine [25,26]

Note: “√” indicates presence of the metabolite; “×” indicates absence of the metabolite.

**Table 4:** Clinical and translational status of metabolomics in infectious diseases.

Domain	Disease/Context	Key Metabolites/Signatures	Current Application Status	Clinical Relevance	Representative References
Routine clinical metabolites (not metabolomics tests)	TB, HIV (research settings)	Kynurenine/Tryptophan ratio	Research and adjunct clinical studies	Immune activation, disease severity	[15,53]
	COVID-19	Bile acids, lipids	Investigational (not routine)	Severity stratification	[54]
Near-clinical/translational metabolomics	Tuberculosis	Kynurenine/Tryptophan ratio	Clinical cohort studies	Treatment response, disease severity	[55]
	COVID-19	Lipidomic and amino-acid signatures	Clinical studies	Prognosis and immune dysregulation	[56]
Vaccine metabolomics (pre-clinical to early translational)	Dengue, Influenza, COVID-19 vaccines	Lipids, amino acids, tryptophan derivatives	Clinical trials only	Early correlates of vaccine responsiveness	[38,57,58,59]

## Data Availability

This is a review article and did not involve data generation.
